# Supplementary material for: Reduced exposure to extreme precipitation from 0.5 °C less warming in global land monsoon regions
Source: Nat Commun. 2018 Aug 8;9:3153. doi: 10.1038/s41467-018-05633-3 (PMC6082837; doi:10.1038/s41467-018-05633-3)
Supplement: Supplementary file 1 — Supplementary Information [file 41467_2018_5633_MOESM1_ESM.pdf]

Supplementary Information for

Reduced exposure to extreme precipitation from 0.5°C less warming in global land monsoon regions

Zhang et al.

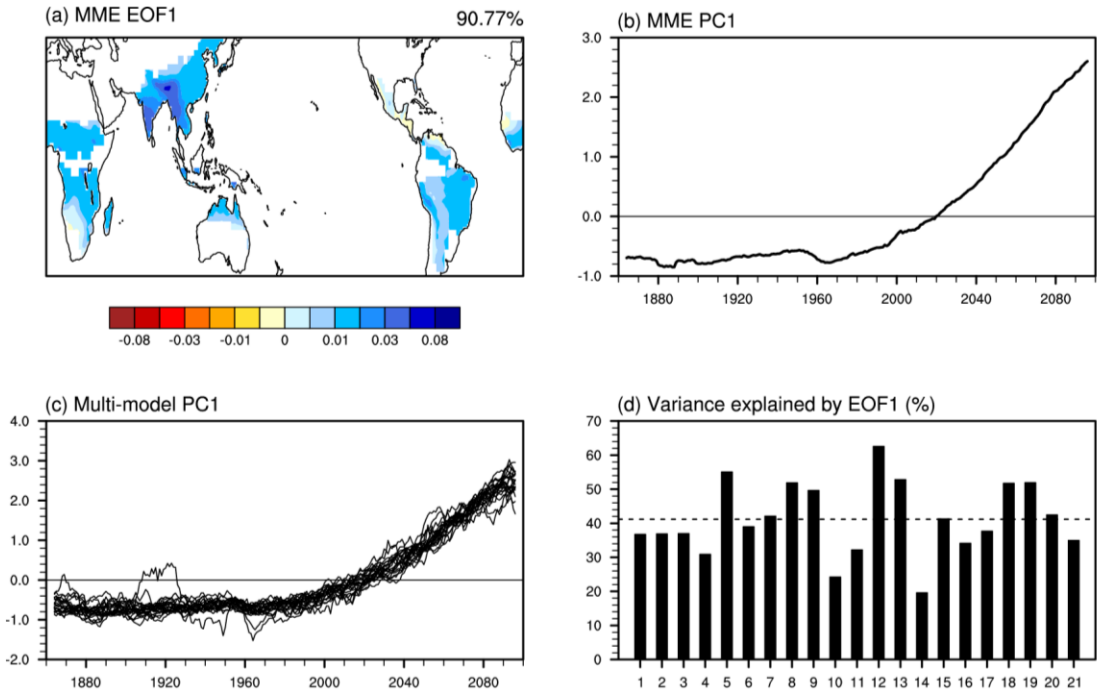

Supplementary Figure 1. The leading mode of EOF for RX5day. (a) The leading mode of the EOF and (b) the corresponding time series of the 9-year running averaged RX5day in the GM region from 1860 to 2100 derived from the multi-model ensemble median (historical + RCP8.5). The variance explained is shown in the top-right. (c) Time series and (d) variance explained by EOF1 of the 9-year running averaged RX5day over the GM region from individual models. The dashed line in (d) indicates the mean of the explained variance across models.

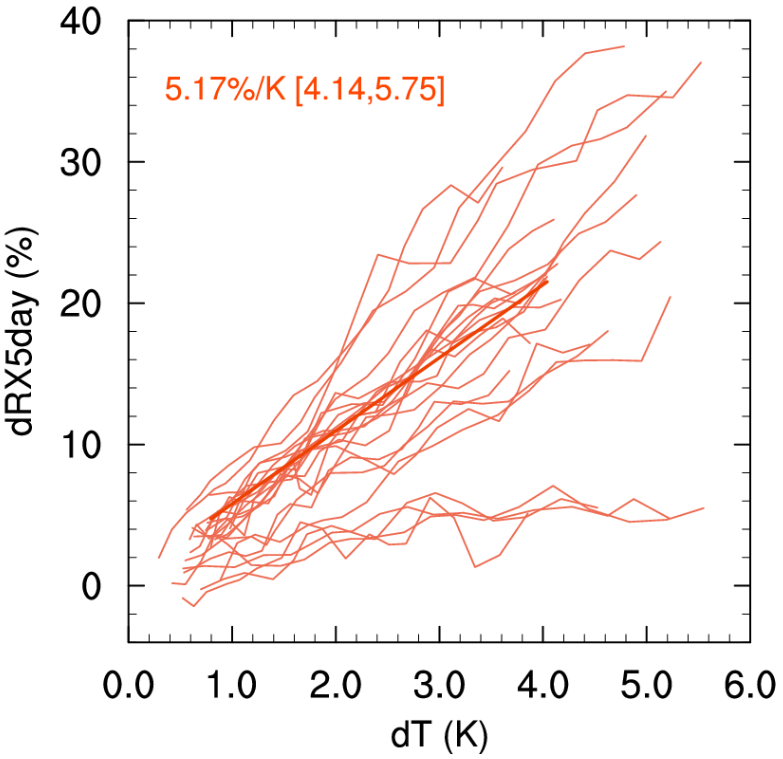

Supplementary Figure 2. Response of RX5day to global warming over the GM region. Changes in RX5day averaged over the GM region versus global mean surface air temperature in the CMIP5 multi-models. Changes are derived from projections under the RCP8.5 over decadal periods starting in 2006 and overlapped by 5 years (i.e., 2006-2015, 2011-2020, up to 2091-2100). Each line represents an individual model, and the thick line denotes linear fitting of the median response. Multi-model median scaling rates (%/K), as well as interquartile ranges, are shown in the top left.

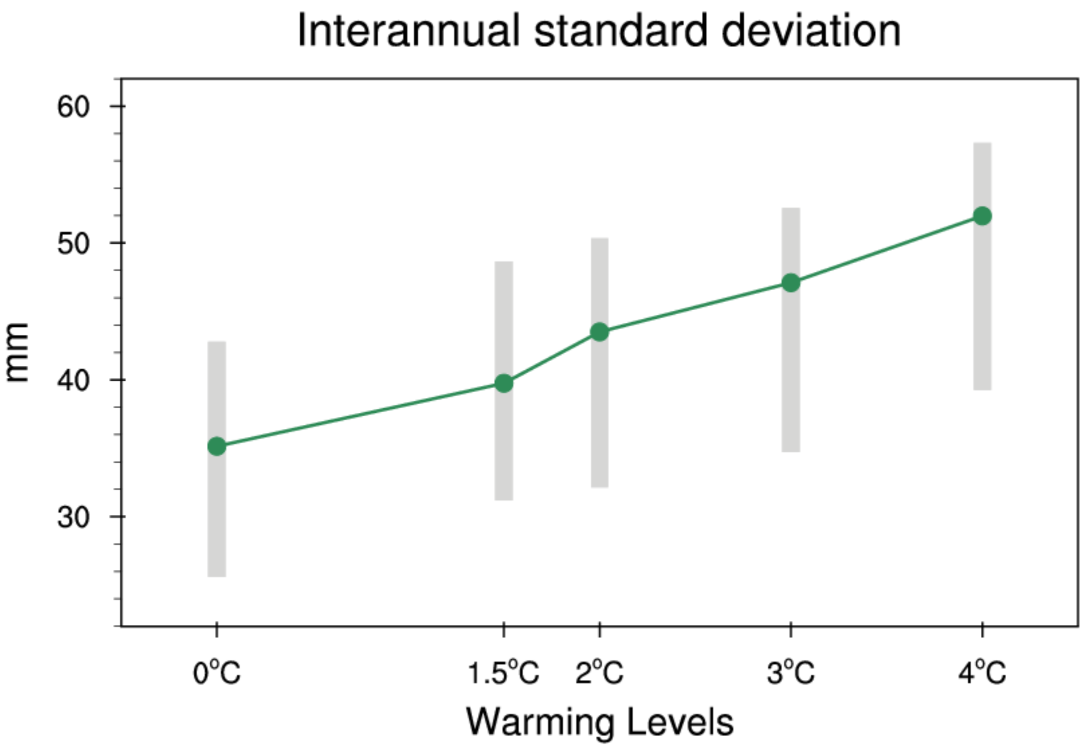

Supplementary Figure 3. Changes in variability of RX5day with global warming. Changes in the interannual standard deviation (units: mm) of RX5day over the GM region at different warming levels under the RCP8.5 projections. Multi-model medians (dots) and 25th-75th ranges (vertical bars) are shown. To calculate the interannual standard deviation, a local detrending with an 11-year running smooth (i.e., removing the 11-year running mean from the original time series) is applied to derive anomalies for each grid box, then the standard deviation is calculated over 20-year segments centered on respective timings. Standard deviations over the GM region are derived with area weighting (see Methods).

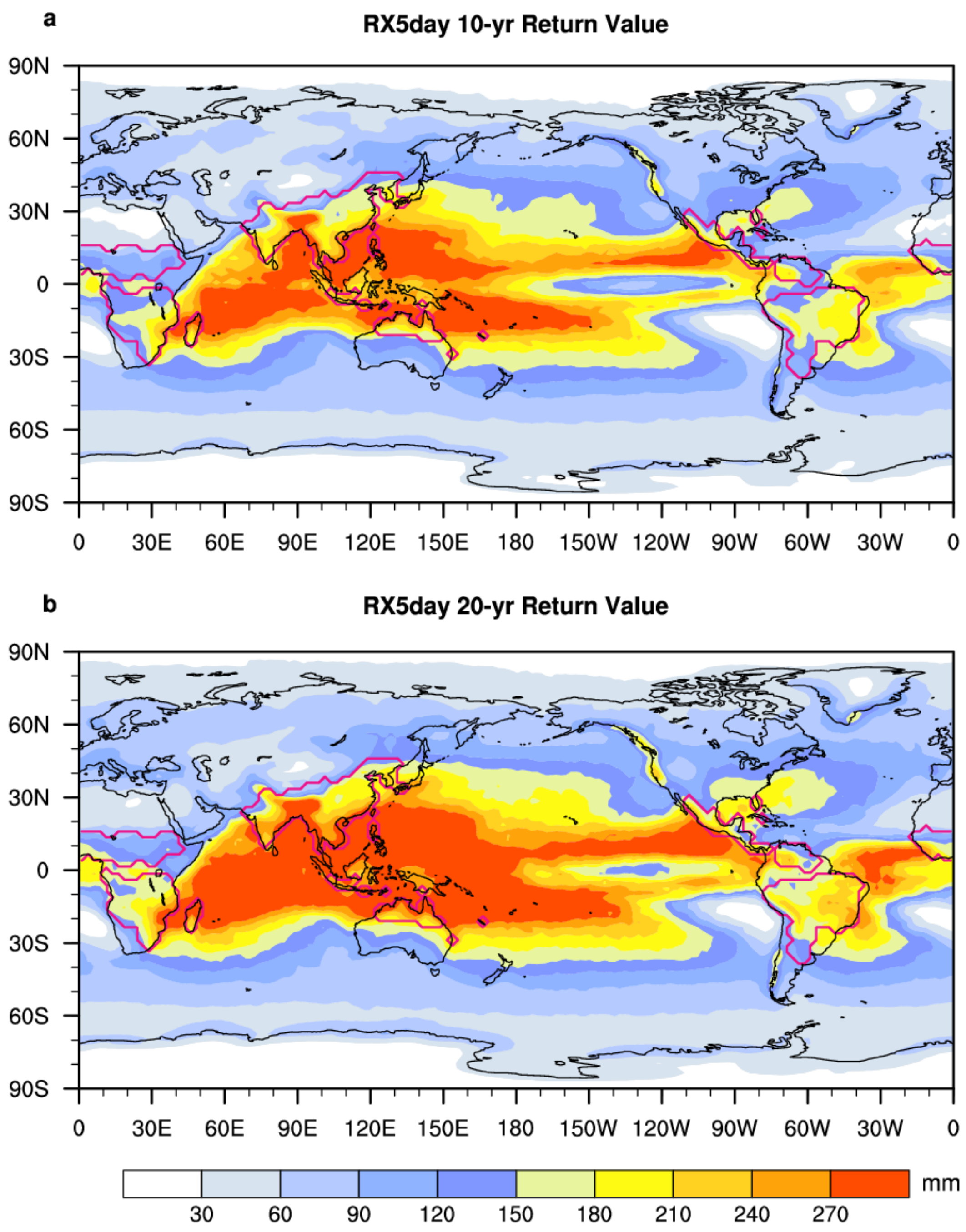

Supplementary Figure 4. Multi-model median 10- and 20-year RX5day return values for the baseline. Multi-model median 10- and 20-year return values (units: mm) from the 1950-2005 baseline, estimated from generalized extreme value (GEV) distributions by the method of maximum likelihood. Magenta lines denote the GM region.

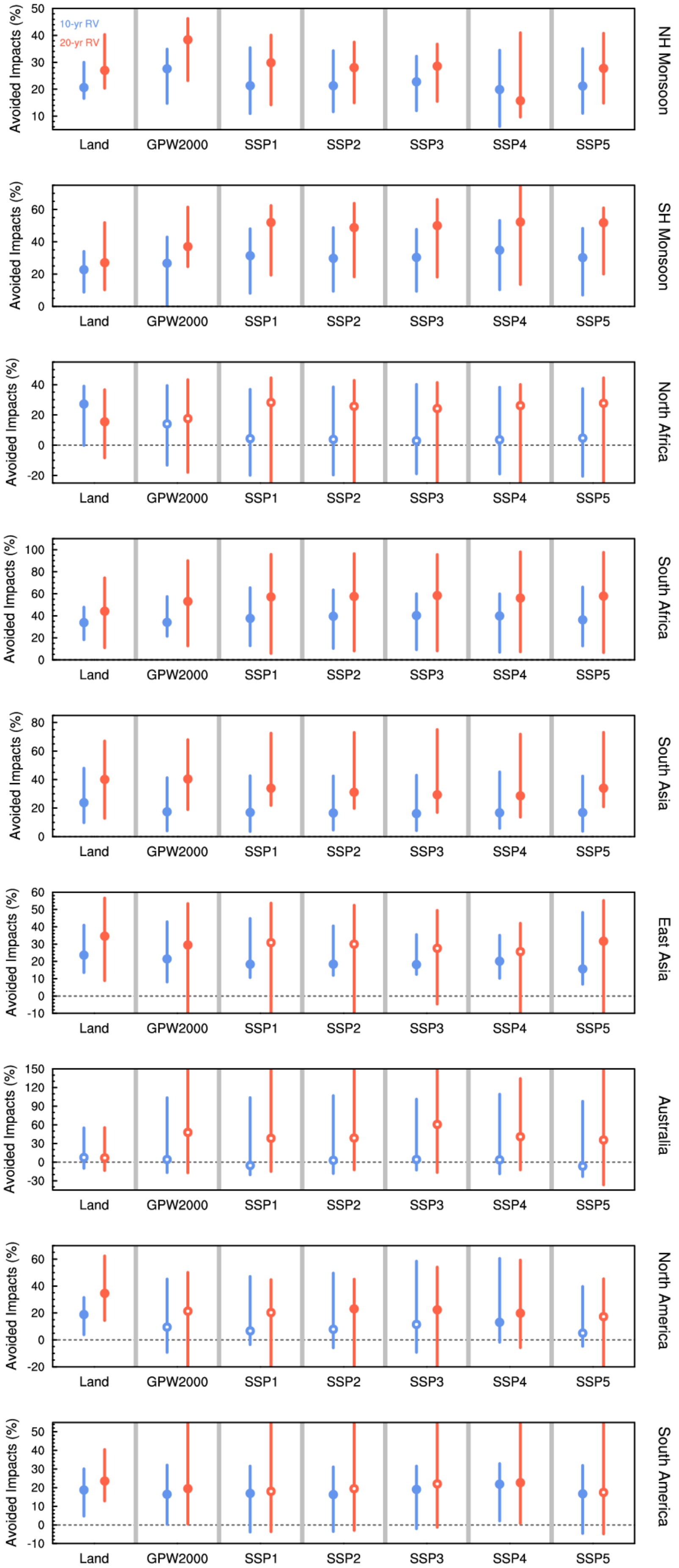

10-yr RV
20-yr RV
Avoided Impacts (%)
Land
GPW2000
SSP1
SSP2
SSP3
SSP4
SSP5
NH Monsoon
SH Monsoon
North Africa
South Africa
South Asia
East Asia
Australia
North America
South America

b

Supplementary Figure 5. Avoided impacts by the half a degree less warming over monsoon sub-regions. Areal and population exposures reduced by the 1.5°C warming compared to 2°C warming for RX5day events that exceed the baseline 10- and 20-year return values. Circles and bars denote multi-model medians and interquartile ranges, respectively. Where more (less) than 2/3 of the models indicate reduced exposure by the 0.5°C less warming are indicated by solid (open) circles.

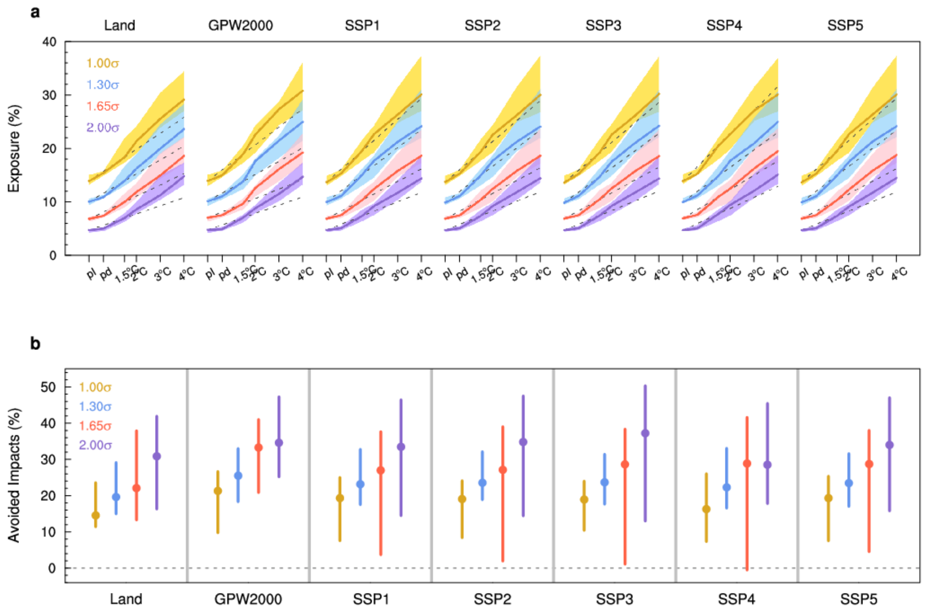

Supplementary Figure 6. Areal and population exposures to dangerous RX5day events over the GM region. a, Fraction of land area and population experiencing RX5day events that exceed 1.00, 1.30, 1.65, and 2.00 σ (inter-annual standard deviation in the 1950-2005 baseline) at the pre-industrial (pI, 1861-1890, 0°C), present-day (pd, 1986-2005), 1.5°C, 2°C, 3°C, and 4°C warming levels, over the GM region (see Methods). Population in 2000 (GPW2000) and under SSPs 1-5 in 2100 are used to estimate population exposure. The multi-model medians (solid lines) and interquartile ranges (shadings) are shown. The abscissa in **a** is proportional to the warming magnitudes, where a warming of 0.61°C is set for 1986-2005. The dashed gray lines denote the linear extrapolation from the pre-industrial (0°C) and 1.5°C warming levels. **b**, Areal and population exposures reduced by the 1.5°C warming compared to 2°C warming for RX5day events that exceed different σ levels (see Methods). Circles and bars denote multi-model medians and interquartile ranges, respectively. Where more (less) than 2/3 of the models indicate reduced exposure by the 0.5°C less warming are indicated by solid (open) circles.

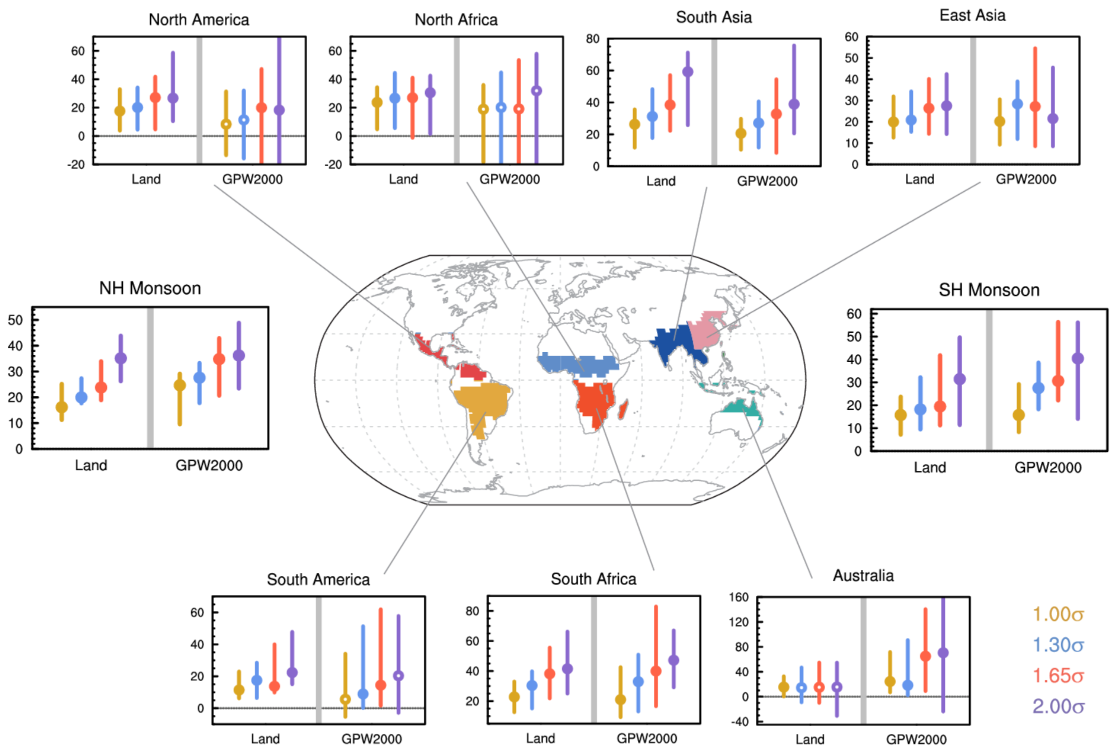

Supplementary Figure 7. Avoided impacts by the half a degree less warming over monsoon subregions. Areal and population exposures (%) reduced by the 1.5°C warming compared to 2°C warming for RX5day events that exceed 1.00, 1.30, 1.65, and 2.00 σ (see Methods). Circles and bars denote multi-model medians and interquartile ranges, respectively. Where more (less) than 2/3 of the models indicate reduced exposure by the 0.5°C less warming are indicated by solid (open) circles. Population exposures based on population in the year 2000 (GPW2000) are shown. Regional monsoon domains are shown in colors on the map.

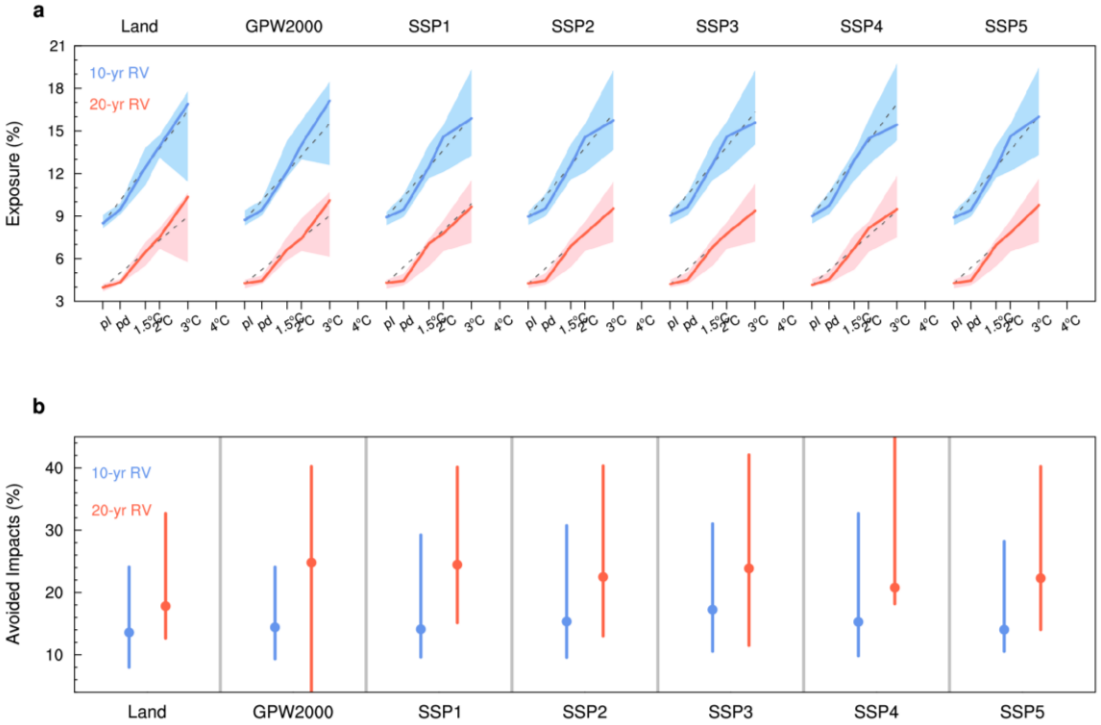

Supplementary Figure 8. Same as Figure 4, but for projections under the RCP4.5 scenario. Note that only 6 out of the 21 models reach a 3°C warming before 2100, while none reach a 4°C warming by 2100.

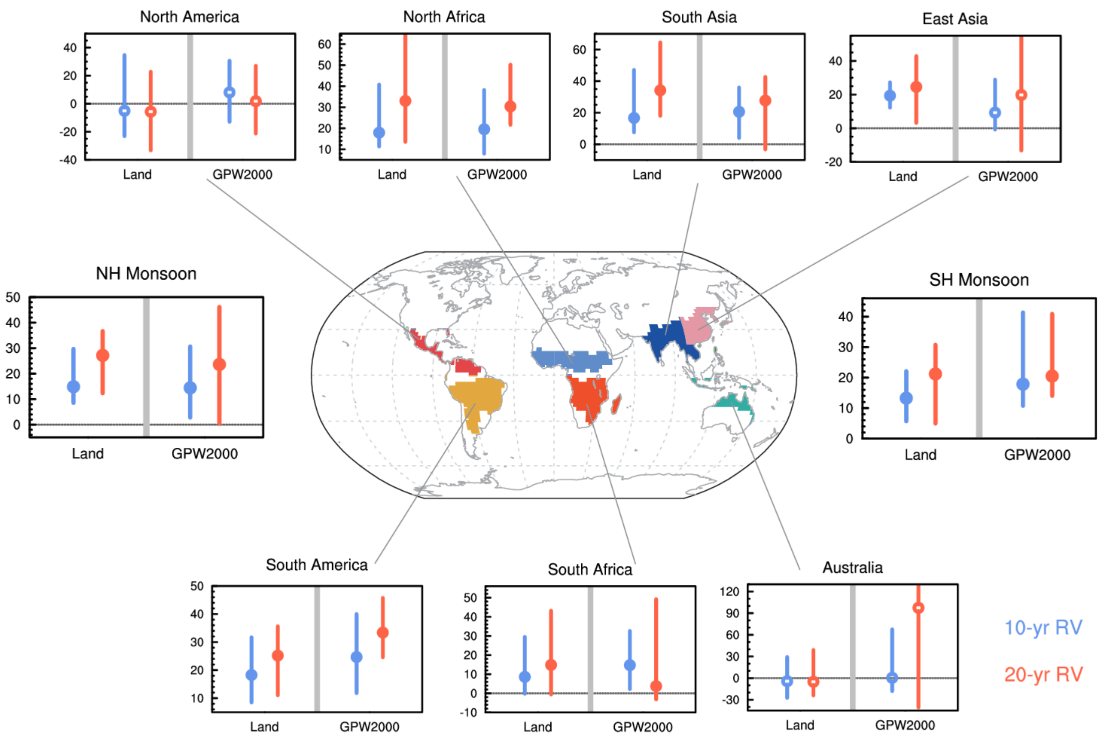

Supplementary Figure 9. Same as Figure 5, but for projections under the RCP4.5 scenario.

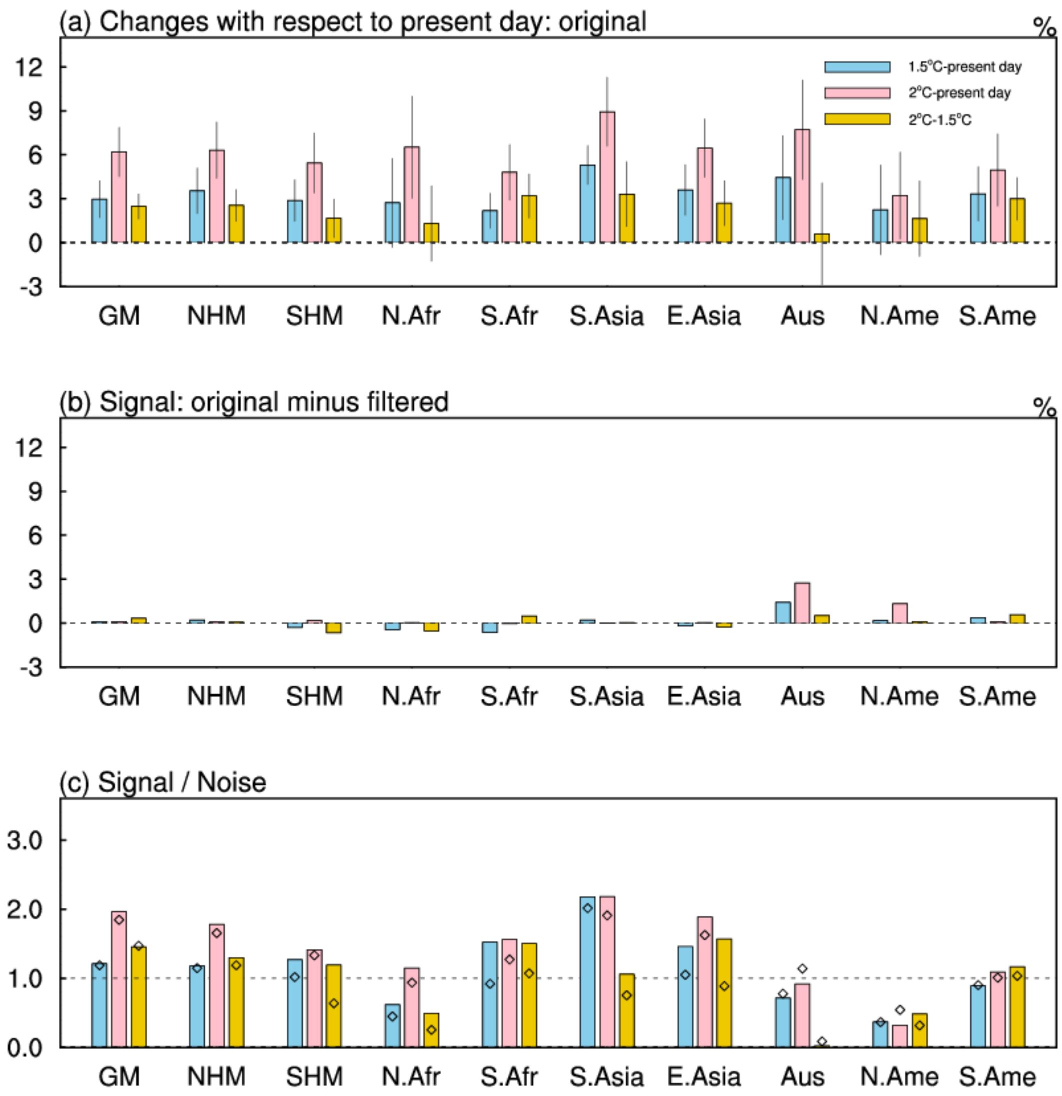

Supplementary Figure 10. (a) Multi-model median changes (histograms; % with respect to the 1986-2005 present-day level) in RX5day for the GM and individual monsoon regions for present day to 1.5°C (blue), present day to 2°C (pink), and 1.5°C to 2°C (green) scenarios, derived from the original time series. Bars denote one standard deviation across models. (b) Differences (% with respect to the 1986-2005 present-day level) in ensemble median changes derived from the original and filtered (by removing the 10-70-year low frequency oscillations) time series. (c) Signal (multi-model median changes) to noise (inter-model standard deviation) ratio (SNR). Histograms represent filtered cases, while diamonds represent original cases.

Supplementary Table 1. List of CMIP5 models used and the timings of 1.5°C, 2°C, 3°C, and 4°C global warming levels above the pre-industrial levels (1861-1890) under the RCP8.5. The symbol "-" indicates that the warming level will not be achieved before 2100. Only the first ensemble member in each model is used.

Model	1.5°C	2°C	3°C	4°C
**ACCESS1-0**	2025	2042	2061	2081
**ACCESS1-3**	2031	2042	2061	2081
**bcc-csm1-1**	2021	2036	2060	2085
**bcc-csm1-1-m**	2012	2028	2061	2086
**CanESM2**	2013	2027	2049	2068
**CCSM4**	2015	2029	2058	2077
**CESM1-BGC**	2017	2034	2059	2081
**CMCC-CM**	2030	2040	2060	2078
**CMCC-CMS**	2029	2042	2062	2077
**CNRM-CM5**	2029	2043	2067	2087
**GFDL-CM3**	2025	2036	2055	2071
**IPSL-CM5A-LR**	2012	2028	2047	2066
**IPSL-CM5A-MR**	2016	2032	2051	2068
**IPSL-CM5B-LR**	2024	2038	2061	2086
**MIROC5**	2035	2050	2073	-
**MIROC-ESM**	2021	2030	2053	2070
**MIROC-ESM-CHEM**	2019	2029	2049	2067
**MPI-ESM-LR**	2015	2036	2061	2082
**MPI-ESM-MR**	2018	2040	2060	2084
**MRI-CGCM3**	2041	2053	2077	-
**NorESM1-M**	2033	2049	2073	-
